# Medicine

**Published:** 1903-06

**Authors:** G. Parker


### Progress of the Medical Sciences.

### MEDICINE.

**Common hemiplegias.**—There is a danger in medicine of our paying attention exclusively to the abstract and rare, and forgetting the common everyday ills of human life, though the latter involve some of the most important problems of pathology. For one sufferer from acromegaly or leukæmia there are a thousand whose lives are cut short or made pitiable wrecks by what the laity call "strokes." Some die at once, some drag out a miserable, drivelling existence, and a few recover to do good work, like Pasteur, for twenty or thirty years, but always with the haunting shadow of a second attack hanging over them. Indeed, to every man in the second half of life, so common is this fate, there comes the fear lest it be his own lot sometime to lie paralysed with broken, muffled speech, and look round sadly on his books and tools which he will never use again, while ruin falls on one faculty after another, the highest first. What are the means of prevention and methods of treatment for these very common hemiplegic paralyses? In these days of collective investigations and crusades against tuberculosis and cancer, there are few subjects more worthy of such a combined effort than this; yet we are apt to pass them by as an inevitable process of decay, partly since they are due to diseases most common in later life, and partly because the pathology is so obscure. But it is not the decrepid only who fall victims. The vigorous, active worker may be struck down in a moment. Even a man with granular kidneys might work and live comfortably for many long years but for a sudden breakdown of his vessels. A convalescent after an illness dies from embolism, or becomes a lacrymose hemiplegic from thrombosis; or, again, a healthy old man after a slight chill or fatigue is attacked with progressive cerebral thrombosis. In certain cases, of course, bacterial infections and the presence of tumours and nerve diseases lead to similar results, but these may to some extent be considered as a separate group. Leaving out for the present these latter, what are the causes of those cerebral hemorrhages, thromboses and emboli? We find certain high tensions, or vascular degenerations, both fibrous and atheromatous, or blood changes, such as increased coagulability, or failing irregular cardiac action, respectively preceding them.

**Forms of increased blood-pressure.**—Besides the cases of high blood-pressure which are connected with renal disease and other known conditions, there are some obscure forms. Graham Steell[1] describes a group of cases most often seen in female patients after the climacteric. The pulse is full between the beats, the tidal wave high and prolonged. Dyspnœa, followed finally by œdema and an enlarged liver, is apt to appear; but the heart is at first neither enlarged or irregular, and there is no murmur. Apparently there may be no kidney disease, but generally much gastric trouble. He advises careful dieting and no farinaceous food after middle day, since the condition depends on a faulty metabolism, which is kept up by ill-digested starchy foods. The discussion started by Clifford Allbutt,[2] on **the rise of blood-pressure in later life,** points to a definite type of disease. He lays down that it exists in two forms: (1) with slack arteries, and (2) with contracted ones; but he thinks that arterio-sclerosis or vaso-constriction are not the chief or primary causes of high pressure even in the latter type. Constriction does not seem to occur in all areas at once, and it is only temporary. He therefore believes that there is some blood change, an increased viscosity which, by the friction it causes, does the work attributed to vaso-constriction. In the long-continued discussion this gave rise to, Broadbent[3]

[1] *Med. Chron.*, 1902-3, 4 s., iv. 161.
[2] *Lancet*, 1903, i. 645. [3] *Ibid.*, 393.

suggested that the increased resistance lay in the capillaries, and that the blood change altered the relation between the blood and their walls; but Allbutt adds that the analogy between these closed vessels and the flow in capillary tubes open to the air is faulty. If the outermost layer of the blood wets the walls of the capillaries, the inner layers slide by on it at a rate depending on their mutual friction or viscosity. Others, again, hold that there is a normal universal tonus or constriction in the arterioles, and that an increase of this causes the high pressure. A. Morison,[1] with a view to solve these questions, asks whether in senile high tension the overgrowth of the connective tissue of the vessels, or the muscular hypertrophy occurs first.

The practical point in Allbutt's address is that we ought to recognise at an earlier stage this rise of pressure, before the whole arterial system has been "strained," and treat it by rigorous diet, suitable exercise and drugs. This recognition can be best made by the sphygmometer, which should not show more than 120 mm. Hg. in a healthy middle-aged man. If treatment is postponed till one is on the verge of apoplexy, relief may be possible, but a cure is not. To discover the early stages of high tension by the finger alone is often difficult. If found it is often put down to Bright's disease, even though definite symptoms are absent, or it is called gout for lack of a better name. Thus he recognises a form of high pressure due to an altered blood state only. This is not caused by, but may lead to, vascular degeneration. Vascular disease, when the result of toxins or of senile decay, does not tend, like this rise of blood-pressure, to hemorrhage, but to thrombotic softening.

As to other causes of excessive tension and their treatment, if kidney disease is present, that must be managed and dangerously high pressures checked. Iodide of sodium may be given for long periods, with intervals of rest. French authorities, in some cases, give a rigid diet of, perhaps, milk every third or fourth week for months or years. Von Noorden,[2] in a most interesting paper, lays down that the amount of fluid taken should be strictly limited, and that a small amount of meat should be allowed regularly, whether red or white meat is immaterial, in chronic kidney disease. When sudden danger from blood-pressure arises, nitrites are, of course, valuable, and their effect is increased, as Lauder Brunton shows, if nitrates are added.

Carter,[3] in recording 500 measurements of pressure by the sphygmometer, finds that sodium nitrite in 2 or 3 grain doses lowers the pressure about 12 mm., but its effects only last one and a half hours. It seems possible, however, that, as in the opposite case of adrenalin, there may be a permanent as well as a temporary action after repeated doses. Of great importance,

[1] *Ibid.*, 614. [2] *Brit. M. J.*, 1902, ii. 1397.
[3] *Am. J. M. Sc.*, 1901, cxxii. 854.

too, are saline purgatives, both to aid elimination and to directly lower pressures. Venesection, with saline injections, is discussed by R. W. Marsden,[1] who takes the view that venesection may relieve an overburdened right heart and temporarily lower pressure, but that if the poison present be an alkaloid, as he thinks it is in the case of uræmia, it is already combined with the tissues and cannot be removed by salines or bleeding. Still, saline injections *per rectum*, or even water at 120° used in the same way, are invaluable diuretics even in uræmic cases. Of course removal of the poison causing the high tension is to be preferred to a direct action on the vascular machinery, because high pressures in themselves may be conservative; but when they reach a danger limit they must be controlled. No one, perhaps, in this country advocates reducing tension by lessening the force of the heart itself, though in America aconite and veratrum are often used.

**Vascular changes.**—As to these, it is useless to say that a man is as old as his arteries, that degenerative changes occur in healthy persons from hereditary causes, and are a natural form of decay. For in the first place most supposed heredities, such as phthisis, turn out to be due to similar surroundings in parent and child, and not heredities at all, so that science has no right to call in heredity as a *deus ex machinâ* to explain facts she has failed to grasp, and, secondly, every workhouse, as Allbutt says, shows plenty of old people with rigid or atheromatous vessels which have not given way. Sometimes these vessels do give way, but not always, and against this we sometimes find **hemorrhages with perfectly healthy vessels and sound hearts**. Endless discussion has taken place on the relation between these vascular changes and high blood-pressures or other blood changes, as to which is the cause of the other, or whether both are effects of a third factor. Degeneration of vessels can hardly be treated while the pathology is so uncertain. Among attempts, however, we may notice Trunicek's serum. He considers that atheroma depends largely on the deposit of calcium phosphate in the vessel walls, and that it is soluble in solutions of sodium chloride, and certain other sodium and magnesium salts. Now, in old age there is a deficiency of sodium chloride in the blood, and he therefore injects subcutaneously a strong solution of these solvent salts. The formula is sodium chloride 10 grammes, sodium sulphate 1, calcium and magnesium sulphate each .75, sodium carbonate .40, sodium phosphate .30; this when mixed is divided into thirteen powders: it may be given by the mouth, rectum, or subcutaneously dissolved in distilled water. Lattes[2] and others report some success from its use in cases of dyspnœa, vertigo, and anginal attacks due to vascular

[1] *Med. Chron.* 1902-3, 4 s., iv. 33.
[2] *Gaz. hebd. de Méd. et de Chir.*, No. 80, 1901; Abstract in *Am. Year Book Med. and Surg.*, 1903.

changes. Rumpf, too, recommends the administration of lactic acid, and the use of a lime-free diet, consisting of meat, fruit, bread, but no rice, milk, eggs, cheese, or cabbage. Haig's dietary may perhaps be considered to act in the same way, as well as in keeping down the pressure.

In syphilis we are upon recognised ground, and the long-continued use of mercury and iodide is known to aid the recovery of damaged vessels.

**Thrombosis** may be due to vascular lesions alone, as in syphilis, or to changes in the blood and vessels, as in septic cases, or to similar changes combined with a failing heart, as in old age. However, the subject was treated in a recent number of this *Journal*, so that only one or two supplementary points need be referred to now. Just as Allbutt insists that the blood-pressure ought to be frequently measured with the sphygmometer, so A. E. Wright[1] advocates the testing of the coagulability where there is any danger of thrombosis. If normal blood takes five minutes to clot, there is no difficulty whatever in finding out whether our patient has blood which under the same conditions clots in one or two minutes. Thus in aged, feeble, or parturient cases, we should estimate the state of the blood as to its readiness to clot just as we test the urine or the heart's action. Moreover, with the apparatus which he recommends, the process can be carried out in a few minutes, and does not require much skill. If excessive coagulability exists, it may be overcome, in many cases, by giving citric acid and the citrates, and the effect measured and charted frequently. He thinks that hemorrhages in typhoid are often due to the deficient coagulability present, and thrombosis in the convalescent stage accompanies an excessive reaction, which is often present then. Indeed, the juice of two lemons is now given daily to typhoid convalescents in one hospital to prevent this disaster. He suggests, too, that the condition, which was very common in the South African war, may be due to the milk diet and out there to the use of concentrated or condensed milk, which contains so many lime salts, milk having more lime than ordinary lime-water has. The existence of such a tendency can only be known by testing, for a very high excess of lime in the blood lowers the coagulability just as a deficiency does. Of course, this leaves out of view other factors in clot formation, such as septic and other changes in the vessel walls and cardiac failure, but it is quite rational to treat each factor as we are able. Moreover, it is curious that thrombosis occurred just in those cases in which he found an excess of coagulability. In aged patients, with irregular hearts, we must insist on the horizontal posture, give cardiac tonics, administer stimulating food in the small hours of the night when the circulation is apt

[1] *Lancet*, 1902, ii. 11.

to fail, and forbid their standing up to pass water, because thrombosis is apt to occur after a fainting attack, or extra exertion, or during sleep. When a thrombus has formed, if the blood state is favourable it may continue to increase, hence the blood should be tested, citrates given freely if indicated, and complete rest with cardiac tonics ordered. James Taylor [1] speaks of the danger of purgatives lest the blood be rendered less fluid. He even gives much liquid by the mouth, when an aperient is needful, to obviate the same risk.

As to **emboli** and their prevention little can be said, except to emphasise the need of rest in diseases in which they are common, especially after second attacks of endocarditis, and during rheumatism, pregnancy, and lactation. In septic cases much may be done, of course, by early treatment of the source to which they are due, as for instance in mastoid disease.

When the pathology of these vascular and blood changes has made greater advances, more efficient means of prevention will be possible, but at present the loss of life and the human misery they produce are a reproach to medical science.

G. PARKER.

[1] Allbutt's *System of Medicine*, vol. vii., 1899, p. 571.

